# Changes in the Dynamics of Foliar N Metabolites in Oak Saplings by Drought and Air Warming Depend on Species and Soil Type

**DOI:** 10.1371/journal.pone.0126701

**Published:** 2015-05-11

**Authors:** Bin Hu, Judy Simon, Madeleine S. Günthardt-Goerg, Matthias Arend, Thomas M. Kuster, Heinz Rennenberg

**Affiliations:** 1 College of Forestry, Northwest A&F University, Yangling, Shaanxi, PR China; 2 Institute of Forest Sciences, University of Freiburg, Freiburg, Germany; 3 Swiss Federal Institute for Forest, Snow and Landscape Research (WSL), Birmensdorf, Switzerland; University of Minho, PORTUGAL

## Abstract

Climate change poses direct or indirect influences on physiological mechanisms in plants. In particular, long living plants like trees have to cope with the predicted climate changes (i.e. drought and air warming) during their life span. The present study aimed to quantify the consequences of simulated climate change for foliar N metabolites over a drought-rewetting-drought course. Saplings of three Central European oak species (i.e. *Quercus robur*, *Q*. *petraea*, *Q*. *pubescens*) were tested on two different soil types (i.e. acidic and calcareous). Consecutive drought periods increased foliar amino acid-N and soluble protein-N concentrations at the expense of structural N in all three oak species. In addition, transient effects on foliar metabolite dynamics were observed over the drought-rewetting-drought course. The lowest levels of foliar soluble protein-N, amino acid-N and potassium cation with a minor response to drought and air warming were found in the oak species originating from the driest/warmest habitat (*Q*. *pubescens*) compared to *Q*. *robur* and *Q*. *petraea*. Higher foliar osmolyte-N and potassium under drought and air warming were observed in all oak species when grown on calcareous versus acidic soil. These results indicate that species-specific differences in physiological mechanisms to compensate drought and elevated temperature are modified by soil acidity.

## Introduction

Current climate change scenarios suggest a significant increase in annual average air temperature (ca. 2.6°C) and significantly reduced summer precipitation by the end of the 21^st^ century compared to 1986–2005 levels in Central Europe [1–4]. Whether woody plants could acclimate to these changes in climate strongly depends on their specific ecological requirements and genetic variability [5]. For example, oak species growing in regions of Europe with relatively dry and warm climate (e.g. *Q*. *pubescens* and *Q*. *suber*) are considered drought- and thermo-tolerant due to their deep penetrating root system, xeromorphic leaf structure and the capacity to interrupt CO_2_ assimilation due to drought and to resume it after rewatering [6–10]. Therefore, these species are expected to withstand or even benefit from future climate change [11–12], whereas other species with relevant shallow rooting systems such as spruce and beech are thought to suffer from impaired water balance [5, 13–15].

Drought and air warming, however, not only affect the water relations of trees, but also their nitrogen balance as indicated by foliar N composition, storage and remobilization [5]. Foliar N storage and remobilization processes respond to drought and enhanced air temperature with compensation mechanisms depending on leaf stomatal conductance and rates of net photosynthesis [5, 16–17]. For example, up-regulation of free amino acid levels in leaves of higher plants is frequently observed and thought to contribute to osmotic adjustment under various stress conditions [18–22]. The reduced cell damage operated by mechanisms of leaf turgor maintenance often occurs at the expense of other leaf N pools, in particular when whole plant N cycling is impaired as a consequence of drought and heat [5, 23]. However, differences among tree species in their capability to mobilize leaf internal N pools for osmotic adjustment by free amino acid accumulation have only recently been elucidated [24–26].

At the end of a “model-ecosystem” experiment with oak saplings exposed to drying-rewetting course for three consecutive years, drought but not air warming caused a strong decline in net photosynthesis and accumulation of amino acids in the leaves at the expense of structural N in the three most abundant European oak species (i.e. *Quercus robur* L., *Q*. *petraea* [Matt.] Libel. and *Q*. *pubescens* Willd.) [9, 25]. Air warming increased stomatal aperture [27] and caused seasonal up-regulation of net photosynthesis [9], but other leaf traits such as composition and contents of non-structural carbohydrates and nitrogen metabolites were not affected [25, 28]. However, physiological differences between oak species were not consistent with their drought tolerance. *Q*. *pubescens*, originating from dry habitats, showed higher drought tolerance compared to *Q*. *robur* regarding growth, but more *Q*. *pubescens* trees were counted with leaf drought injury symptoms in their foliage (leaf margin necrosis and yellowing) than *Q*. *petraea*, originating from wet and moderately dry habitats [10]. Net photosynthesis of the three oak species was similar under well-watered conditions [29].

In the present study, we focused on the development of leaf nitrogen metabolite levels in response to a soil drying-rewetting-drying course with and without air warming in leaves of the three most abundant European oak species (i.e. *Quercus robur* L., *Q*. *petraea* [Matt.] Libel. and *Q*. *pubescens* Willd.). Building on a previous studiy which reported other results of these treatments [25], we hypothesize that (і) effects of drought on foliar N composition at the end of a 1^st^ drought period (1^st^D) were compensated by rewetting, whereas the 2^nd^ period of drought (2^nd^D) would generate significant treatment effects on foliar N composition; (ii) drought and air warming would increase foliar concentrations of organic substances for osmotic adjustment, such as the selected osmolyte-N (reflected by free amino acids accumulation), and inorganic osmolyte cations such as potassium; (iii) foliar N dynamics during a drying-rewetting-drying course would be modified by the soil type.

## Materials and Methods

### Ethic statement

Field sampling in this study was permitted by Swiss Federal Research Institute for Forest, Snow and Landscape Research (WSL). The study did not involve endangered or protected species.

### Experimental design and oak sapling establishment

The present study was part of the interdisciplinary oak research project “Querco” which was initiated in 2003 and experimentally carried out 2006 to 2009 in the research model ecosystem facility of the Swiss Federal Institute for Forest, Snow and Landscape Research (WSL) in Birmensdorf, Switzerland (545 m a.s.l., 47°21'48'' N, 8°27'23'' E). Experimental design and treatment details have previously been reported [8–9, 10, 30]. A total of 16 large (3m height, 6.7 m^2^ area) hexagonal ecosystem chambers were arranged in a Latin square with four repetition chambers for each treatment. The four climate treatments were (i) control (CO, with ambient conditions), (ii) increased air temperature = air warming (AW), (iii) reduced irrigation = drought (D), and (iv) air warming and drought (AWD). The four climate treatments were applied during the growing season from April to October each in 2007, 2008 and 2009. Natural rainfall was excluded from the chambers by automatic closure of the glass roofs at the beginning of rainfall events. During winter between the growing seasons, the roofs were kept open to allow for natural rainfall exposure. Drought periods were artificially applied by stopping irrigation for several consecutive weeks during selected periods. Air temperature was passively increased by reducing the opening angle of the chamber glass side walls. Each chamber was split into two 1.5 m height concrete-walled soil lysimeter compartments and filled in spring 2005 with either calcareous Fluvisol (sandy loam, pH 6.9) or acidic Haplic Alisol (loamy sand, pH 4.0) to 1 m depth with soil originating from mixed natural oak stands. Underneath the soil layers, a 0.5 m pure quartz gravel drainage stratum was established. The soils were selected for different acidity with otherwise similar properties (for details on soil physical and chemical properties see [30]).

The three oak species (i.e. *Quercus robur* L., *Q*. *petraea* [Matt.] Liebl. and *Q*. *pubescens* Willd.) were represented in the experiment by 4 provenances each. Acorns collected in 2003 originated from ‘natural’ oak stands located in the Swiss midland, western (Jura) and southern Switzerland, representing the Swiss distribution of the species (for details on plants and planting see [10], for genetic information see [30]). For the present study, the midland provenance was selected for *Q*. *robur*, the Jura provenance for *Q*. *petraea* and the southern Switzerland provenience for *Q*. *pubescens*, according to main territories of distribution (for details of the provenance sites see [8]).

The present study has been carried out during the drought treatments in the year 2009 with two prolonged water deprivation periods plus one short rewetting period in between for sapling recovery. This led to minimum soil water levels of less than 0.05 m^3^ m^-3^ in the entire calcareous soil profiles at the end of the first and during the second drought period in 2009 [30]; periods of drought < 0.06 m^3.^m^-3^ are shown in Table 1. The first prolonged period without irrigation (= 1^st^D) took place from 10^th^, March until 30^th^, June 2009 (113 days) and the second drought period (= 2^nd^D) from 7^th^, July to 20^th^, August 2009 (45 days) with a short rewetting period in between (= RW) from 1^st^ to 6^th^, July (6 days) [30]. The irrigation of the control chambers remained 30% higher in 2009 compared to the long-term average of the local mean ambient precipitation (728 mm, from April to October, 1963 to 2009). Drought treatments had 43% lower irrigation than the mean local precipitation [30]. Mean predawn water potentials in the leaves of drought-exposed saplings decreased to a level between -1.9 and -2.6 MPa by the end of the 1^st^D period (minimum to maximum average over both soil types and D & AWD of the three species) and between -2.9 and -3.4 MPa at the end of the 2^nd^ D period [10]. For the enhanced air temperature treatment, average daytime air temperature inside the chambers was increased by 1 to 2°C (Table 1), and average soil temperature at a depth of 12 cm increased by 0.5 to 1°C compared to the controls during the growing season of 2009 [30].

**Table 1:** Drought periods and air warming.

Treatment	Drought (weeks)^1^			
	the 1 ^st^ D		the 2 ^nd^ D	
	**acidic soil**	**calcareous**	**acidic**	**calcareous**
**D**	2.5 ± 0.8	4.6 ± 0.8	1.7 ± 0.4	3.1 ± 0.4
**AWD**	1.7 ± 0.5	7.2 ± 0.8	1.6 ± 0.3	3.4 ± 0.4
	**Air warming** ^**2**^			
	**April**		**May—September**	
**AW**	+ 2.0°C		+ 1.1°C	

^1^Mean number of weeks (± SE) per drought period with a continuous soil water content < 0.06 m^3.^m^-3^ at a soil depth from 0 to 25 cm in the D and AWD treatment, see [10] for details.

^2^Warming of daytime air temperature (8:00–18:00 h, UTC+1) (compared to the control), details see [30].

### Leaf sampling

Leaf samples were collected at three different dates during the growing season of 2009, i.e. at the end of the 1^st^D period (30^th^, June), shortly (8 days) after the RW period (14^th^, July) and at the end of the 2^nd^D period (20^th^, August). For biochemical analyses, five to six leaves with approximately 1.5–2 g total fresh weight were taken from each tree, with four oak tree repetitions for each species, climate treatment, and soil type at each harvest. All leaf samples were immediately shock-frozen in liquid nitrogen and stored at -80°C until further analysis. Prior to biochemical analyses, all leaf samples were ground to a homogeneous fine powder in liquid nitrogen.

### Biochemical analyses

#### Quantification of foliar total carbon and nitrogen concentrations

Foliar total carbon and nitrogen contents were determined as described by [31–32]. The dried leaf material (at 60°C for 48 h) with aliquots of 2.0–2.5 mg were weighed into tin capsules (IVA Analysentechnik, Meerbusch, Germany) and analyzed using a C/N elemental analyzer (NA 2500, CE Instruments, Milan, Italy) coupled by a Conflo II interface (Thermo Finnigan MAT, Bremen, Germany) to an isotope ratio mass spectrometer (Delta plus, Thermo Finnigan MAT, Bremen, Germany).

Fine grounded dry leaf powder (Retsch MM2000 zirkonoxid-bowl ultra-centrifuge mill), was digested in a high-pressure microwave system (UltraClav by Milstone: 240°C, 12MPa) and potassium was analyzed in duplicate (range < 10%) by ICP-OES (Optima 7300DV by Perkin Elmer) at WSL central laboratory, according to ISO 17025.

#### Quantification of foliar total soluble protein concentrations

Total soluble proteins were extracted from finely grounded frozen homogenized leaf powder and quantified using a modified version of the method described by [32–33]. To remove phenolic constituents, approximately 0.05 g were weighed and mixed with approximately 0.15 g polyvinylpolypyrrolidone (PVPP 6755, Sigma-Aldrich, Steinheim, Germany). For the extraction of soluble proteins, 1.5 mL buffer containing 50 mM Tris-HCl (pH 8.0), 1 mM ethylenediaminetetraacetic acid (EDTA), 15% glycerol (v/v), 100 mM phenylmethysulfonyl fluoride (PMSF), 1 M dithiothreitol (DTT) and 1‰ Trition X-100 were added. After incubation for 30 minutes at 4°C, samples were centrifuged at 14,000 *g* for 10 min at 4°C. A total of 500 μL 10% trichloroacetic acid was added to an aliquot of 500 μL of the supernatant followed by 10 min incubation at room temperature for protein precipitation. After 10 min centrifugation at 14,000 *g* and 4°C, the pellets were dissolved in 1 mL 1M KOH. Protein concentrations were quantified by adding 200 μL Bradford reagent (Amresco, Solon, Ohio, USA) to 5 μL aliquots of the extracts in 96-well micro-titer plates (Sarstedt, Newton, North Carolina, USA). All samples were measured in three technical replicates. Optical density was determined with a micro-plate spectrophotometer (Tecan, Groedig, Austria) at 595 nm wavelength. Bovine serum albumin (BSA A-7030, Sigma-Aldrich, Munich, Germany) was used as standard.

#### Quantification of total free amino acid concentrations in the leaves

Foliar amino acids were extracted using the method described by [34]. For this purpose, approximately 0.05 g finely homogenized frozen leaf powder were added to 1 mL methanol:chloroform (3.5:1.5, v:v) and 0.2 mL Hepes-buffer (containing 20 mM Hepes, 5 mM ethylene glycol tetra acetic acid (EGTA) and 10 mM NaF, pH 7.0). After 30 min incubation on ice, water-soluble amino acids were extracted twice by adding 600 μL distilled water each time; the suspension was vortex briefly and centrifuged for 5 min (14,000 *g*, 4°C). The combined supernatants were transferred to new tubes and stored on ice. Total amino acid concentrations were quantified from the combined supernatants using a modification of the method described by [32]. For this purpose, aliquots of 100 μL extract plus 100 μL ninhydrin reagent (1:1 mixture of solution A containing 4.2 g monohydrate citric acid and 0.134 g anhydrous stannous chloride in 40 ml 1M NaOH, made up to 100 ml with distilled water (pH 5.0) and solution B containing 4 g ninhydrin in 100 mL ethylene-glycol-monomethylether) were boiled at 100°C for 30 minutes. Isopropanol (1.25 ml, 50%) was added to the samples followed by 15 min incubation at room temperature in the dark. The optical density was determined using a UV-DU650 spectrophotometer (Beckman Coulter, Fullerton, CA, USA) at 570 nm wavelength. L-glutamine was used as standard.

#### Quantification of specific free amino acid compounds and ammonium concentrations in the leaves

Amino acids and ammonium were extracted as described above and the combined supernatants were freeze-dried and re-dissolved in 0.5 mL of 0.02 M HCl. Derivatization and quantification of specific amino acid compounds was performed as described by [35]. For derivatization, 5 μL of the re-dissolved extracts were mixed with 35 μL of AccQ-Tag Ultra Borate buffer and 10 μL of AccQ-Tag Reagent (both Waters, Milford, MA, USA). The samples were derivatized for 10 min at 55°C after incubation for 1 min at room temperature. An internal standard (Norvaline; 10 μM in reaction mixture) was added to the AccQ-Tag Ultra Borate buffer to account for differences in the efficiency of derivatization between samples. Determination of the composition and concentrations of specific amino acid compounds and ammonium were performed with an ultra-performance liquid chromatography system (Waters, Milford, MA, USA) equipped with an AccQ-Tag Ultra column (2.1 × 100 mm; Waters, Milford, MA, USA). Standard H of amino compounds (NCI0180, Pierce, Rockford, IL, USA) was used as analytical standard with amino compounds and ammonium contents added according to the composition of the leaf samples.

#### Quantification of foliar nitrate concentrations

Foliar nitrate contents were quantified using the method described by [32]. For extraction, approximately 0.05 g of frozen homogenized leaf material was added to 0.1 g polyvinylpyrrolidone (PVPP 6755, Sigma-Aldrich, Steinheim, Germany) soaked in 1 mL distilled water for 2 h. After shaking in the dark at 4°C for 1 h, extracts were boiled at 100°C for 10 min and centrifuged (14,000 *g*) at 4°C for 10 min. Nitrate in the supernatant was determined with an ion chromatograph (DX 120, Dionex, Idstein, Germany) coupled to an auto-sampler (AS 3500, Thermo Separation Products, Piscataway, NJ, USA). The ion chromatograph was equipped with a guard column (RFIC Ionpac AG9-SC, 4 × 50 mm, Dionex, Sunnyvale, CA, USA), an analytical column (IonPac AS9-SC, 4 × 250 mm, Dionex, Sunnyvale, CA, USA) and a self-regenerating suppressor (ASRS-ULTRA II, 4 mm, Dionex, Sunnyvale, CA, USA). Anion mixtures of NO_3_^-^, PO_4_^3-^, SO_3_^2-^, and SO_4_^2-^ in distilled water were used as standards [25]. Foliar nitrate levels detected in the present study were extremely low (ca. 4.43 ± 0.55 μg NO_3_^-^ -N g^-1^ DW) compared to other N compounds as also observed by [36].

#### Calculation of foliar structural N concentrations

Foliar structural N contents were calculated by subtracting the N fractions in total soluble proteins and total amino acids from total nitrogen. Calculations were based on the dry weight (DW) of the samples. The ammonium-N contents in oak leaves determined by UPLC analyses and the nitrate concentrations determined by ion chromatography were found to be negligible; therefore, ammonium and nitrate contents were not considered for the calculation of foliar structural N in the present study.

### Statistical analysis

All statistical analyses in the present study were performed using SAS 9.1 (Institute Inc., Cary NC, USA), SPSS 20.0 (SPSS, Chicago, IL, USA) and Sigmaplot 11.0 (Systat Software, Erkrath, Germany). First, either Kolmogorov-Smirnov or Shapiro-Wilk tests were performed to test for normal distribution. Analysis of variance (ANOVA) was performed to determine the main effects. For the treatment effects, the main factors AW, D and their interaction were tested against "chamber mean squares" (error term = interaction row*column*AW*D), according to the Latin square arrangement of the chambers. The effects of soil type with interaction with the treatments were tested against "subplot" mean squares (error term = interaction row*column* AW*D*soil) and the effects of the drought periods and their interactions were tested as nested within the subplot mean squares (error term = row*column*AW*D*soil*period). Significant differences (i) across climate treatments within each species, the drought-rewetting-drought course and soil type, (ii) across species within each climate treatment, the drought-rewetting-drought course and soil type, (iii) across the drought-rewetting-drought course within each climate treatment, species and soil type were tested using *post-hoc* Bonferroni multiple comparison tests (*β* = *α*/n, *α* = significance level of 0.05; n = number of tests). Pair-wise *t*-tests were used to detect significant differences between the two soil types within each climate treatment, species and the drought-rewetting-drought course. Differences were considered significant at *P* ≤ 0.050. All statistical tests were based on four independent repetitions of oak saplings (individual trees) for each climate treatment, species, time point within the drought-rewetting-drought course, and soil type.

## Results

### Responses of foliar total C and N concentrations and the C:N ratio to the treatments

**Fig 1:**
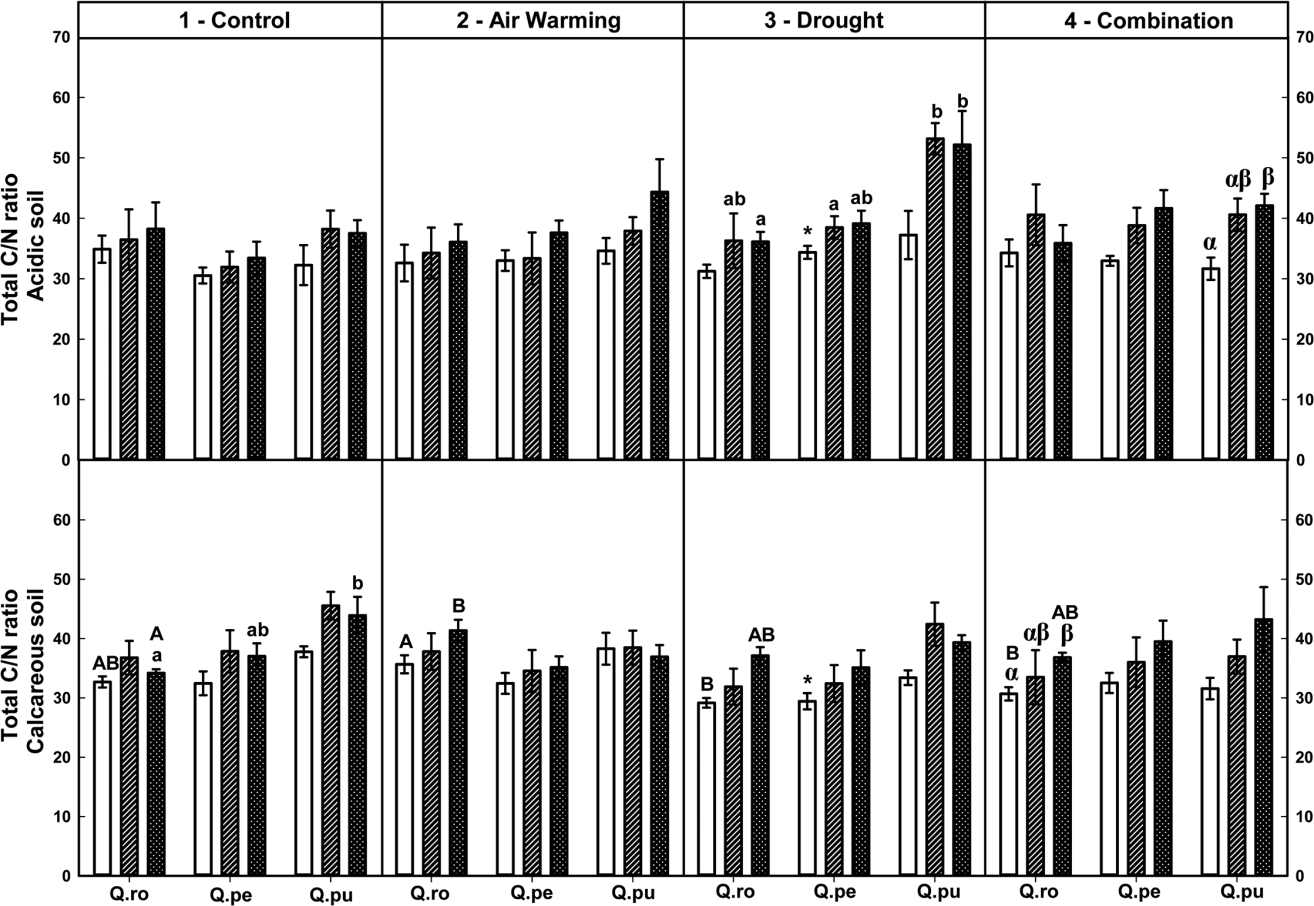
C:N ratio in leaves of three oak species exposed to different climate treatments and soil types during a drought-rewetting-drought course. Vertical bar charts show mean and standard error and the bar charts were grouped for each species, in each treatment and soil type. Periods are indicated from left to right side: white bar = the first drought period (1^st^D), hedged bar = the rewetting period (RW), cyclone bar = the second drought period (2^nd^D). Different small letters indicate significant differences across three oak species within each climate treatment, period and soil type. Different capital letters indicate significant differences across different climate treatments for each species, period and soil type. Greek symbols indicate significant differences across the different drought-rewetting-drought periods for each species, climate treatment and soil type. Asterisks indicate significant differences between acidic and calcareous soil within each climate treatment, species and time course (*P* ≤ 0.050).

**Fig 2:**
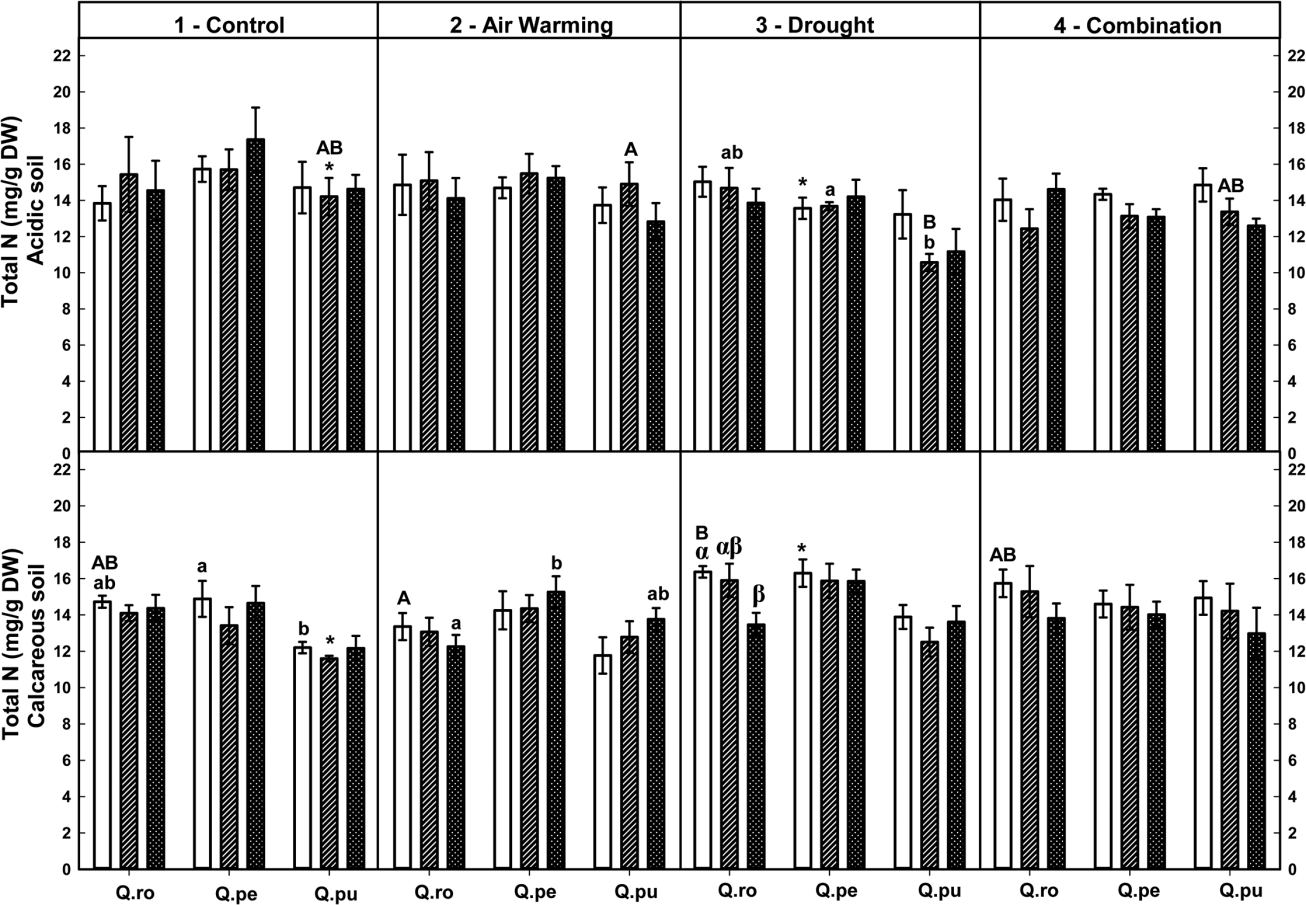
Foliar total N of three oak species exposed to different climate treatments and soil types during a drought-rewetting-drought course. Vertical bar charts show mean and standard error and the bar charts were grouped for each species, in each treatment and soil type. Periods are indicated from left to right side: white bar = the first drought period (1^st^D), hedged bar = the rewetting period (RW), cyclone bar = the second drought period (2^nd^D). Different small letters indicate significant differences across three oak species within each climate treatment, period and soil type. Different capital letters indicate significant differences across different climate treatments for each species, period and soil type. Greek symbols indicate significant differences across the different drought-rewetting-drought periods for each species, climate treatment and soil type. Asterisks indicate significant differences between acidic and calcareous soil within each climate treatment, species and time course (*P* ≤ 0.050).

**Fig 3:**
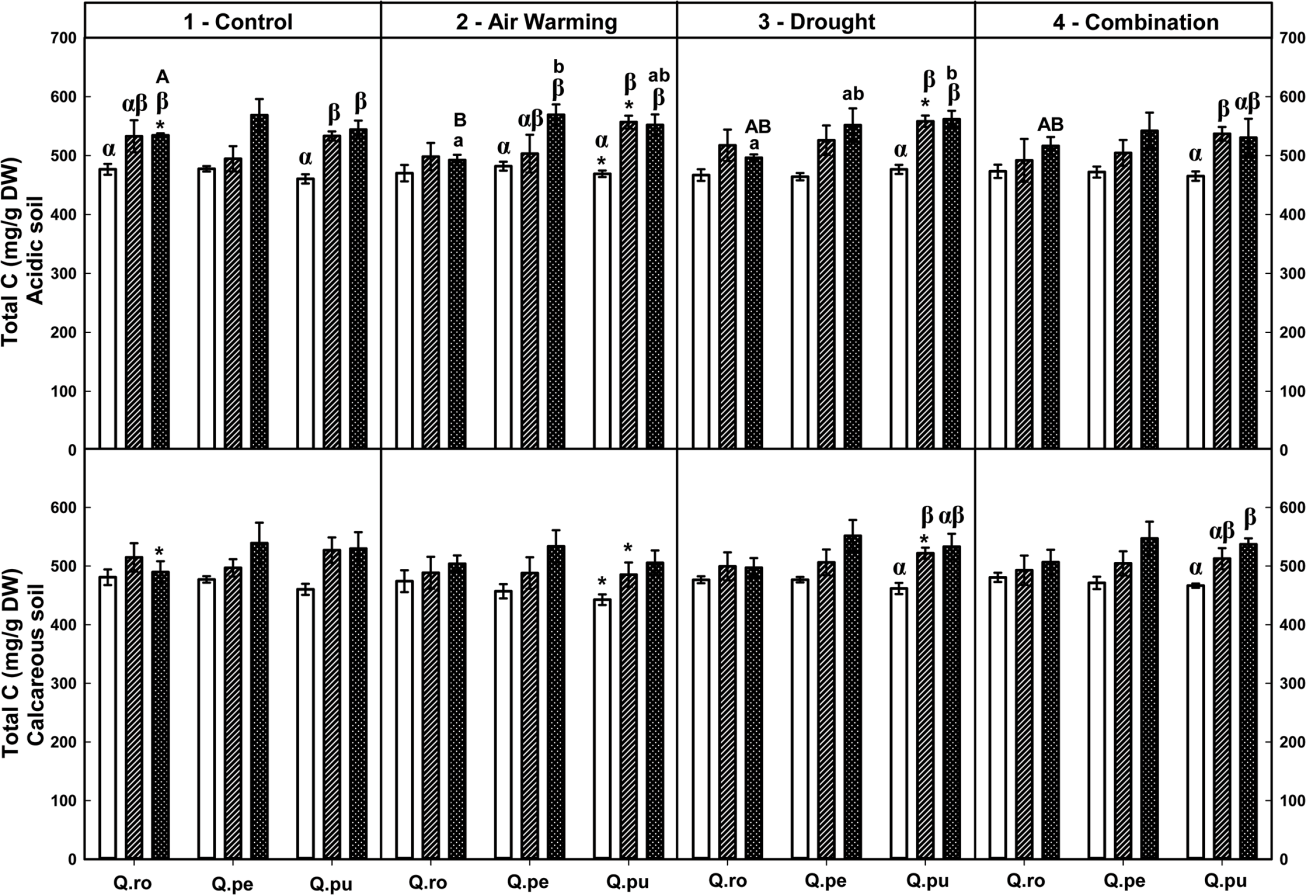
Foliar total C of three oak species exposed to different climate treatments and soil types during a drought-rewetting-drought course. Vertical bar charts show mean and standard error and the bar charts were grouped for each species, in each treatment and soil type. Periods are indicated from left to right side: white bar = the first drought period (1^st^D), hedged bar = the rewetting period (RW), cyclone bar = the second drought period (2^nd^D). Different small letters indicate significant differences across three oak species within each climate treatment, period and soil type. Different capital letters indicate significant differences across different climate treatments for each species, period and soil type. Greek symbols indicate significant differences across the different drought-rewetting-drought periods for each species, climate treatment and soil type. Asterisks indicate significant differences between acidic and calcareous soil within each climate treatment, species and time course (*P* ≤ 0.050).

Overall, the strongest effects on foliar total C and the C:N ratio but not on foliar total N occurred by the drought-rewetting-drought course in all treatments (Table 2, Figs 1–3). Moreover, foliar total C concentrations of all oak species were on average increased after RW and the 2^nd^D period compared to the 1^st^D period regardless of treatment and soil type (*P* ≤ 0.035, Fig 3). Effects on foliar N concentrations as well as C:N ratio were not changed by the soil type at the end of the 2^nd^D period (Table 2, Figs 1 and 2).

**Table 2:** Main effects table during the experimental periods of 2009.

**Factor**	**Total N**	**Total C**	**C:N**	**Total protein-N**	**Total amino acid-N**	**Structural-N**
	**Changes**					
**Species(spec)**	*Qpu*<*Qro* = *Qpe* ^***^	*Qro*<*Qpe* = *Qpu* ^**^	*Qro* = *Qpe*<*Qpu* ^***^	*Qpu*<*Qpe*<*Qro* ^***^	*Qpu* = *Qpe*<*Qro* ^***^	*Qpu* = *Qro*<*Qpe* ^***^
**Period**	ns	1<2<3^***^	1<2 = 3^***^	2<1<3^***^	2 = 1<3^***^	3<2 = 1^***^
**AW**	ns	-1.4^**^	ns	ns	ns	ns
**D**	ns	ns	ns	+20.6^**^	+48.3^***^	-8.4^*^
**Soil**	ns	-2.3^**^	ns	+9.6^**^	+15.2^***^	ns
**Significant interactions**	spec*AW,period*D,D*soil	spec*period	spec*AW,D*soil	spec*period,spec*D,spec*soil,period*AW,AW*D	spec*period,spec*D,period*D,period*soil,AW*D,D*soil	spec*period,spec*AW,period*D,D*soil
**Factor**	**Potassium (K)**	**Gln-N**	**Gly-N**	**Glu-N**	**Asp-N**	**Ser-N**
	**Changes**					
**Species(spec)**	*Qpu* = *Qpe*<*Qro* ^***^	*Qpe* = *Qpu*<*Qro* ^***^	ns	*Qpu* = *Qpe*<*Qro* ^**^	ns	ns
**Period**	na	1 = 2<3^***^	2 = 3<1^***^	ns	ns	ns
**AW**	ns	ns	+19.6^*^	+18.6^(*)^	+25.9^*^	ns
**D**	-10.0^**^	+237.3^***^	+18.6^*^	+53.7^**^	+33.6^*^	+30.1^**^
**Soil**	+16.5^**^	ns	ns	ns	ns	ns
**Significant interactions**		spec*period,spec*D,period*D,period*soil	spec*soil,AW*soil	spec*period,period*soil,AW*D	spec*period,AW*D,D*soil	spec*D,spec*soil

Difference among species, drought-rewetting-drought periods (1 = at the end of 1^st^D period, 2 = after RW period and 3 = at the end of 2^nd^D period) or differences versus control (± %) or calcareous versus acidic soil (± %); significant difference:

(*) *P* ≤ 0.1,

* *P* ≤ 0.05,

** *P* ≤ 0.01,

*** *P* ≤ 0.001;

ns = not significant *P* > 0.05;

na = not available, because foliar K concentrations were determined only at the end of 2^nd^D period.

Particular results show that at the end of the second drought period (2^nd^ D), only Air warming (AW) led to increased foliar C:N ratios in *Q*. *robur* (*Qro*) on calcareous soil compared to the control (*P* = 0.009, Fig 1). At the end of the 2^nd^D period, the AWD treatment led to increased total C:N ratios in the leaves of *Qro* and *Q*. *pubescens (Qpu* compared to the 1^st^D period (*Qro* calcareous soil, *Qpu* acidic soil, *P* ≤ 0.025, Fig 1). Across species under D on acidic soil, *Qpu* had enhanced foliar total C:N ratios compared to *Q*. *petraea (Qpe)* (after RW) and *Qro* at the end of the 2^nd^D period (*P* = 0.013 and 0.034, respectively; Fig 1). In contrast, foliar total C concentration of *Qro* on acidic soil was significantly lower compared to *Qpu* (D) and *Qpe* (AW) at the end of the 2^nd^D period (*P* = 0.035 and 0.019, respectively; Fig 3).

### Effects of drought and air warming on foliar N composition and potassium

**Fig 4:**
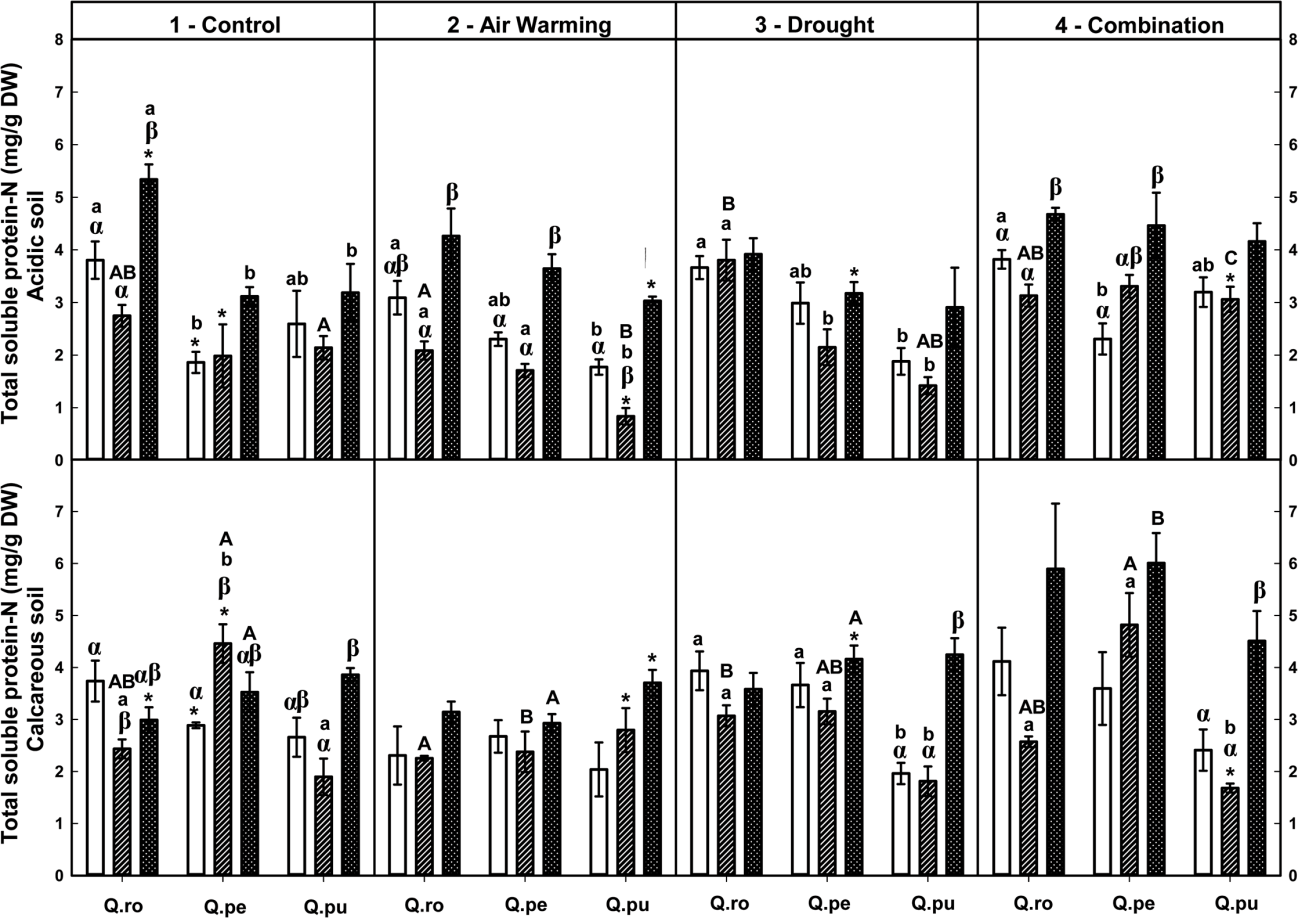
Foliar total soluble protein-N of three oak species exposed to different climate treatments and soil types during a drought-rewetting-drought course. Vertical bar charts show mean and standard error and the bar charts were grouped for each species, in each treatment and soil type. Periods are indicated from left to right side: white bar = the first drought period (1^st^D), hedged bar = the rewetting period (RW), cyclone bar = the second drought period (2^nd^D). Different small letters indicate significant differences across three oak species within each climate treatment, period and soil type. Different capital letters indicate significant differences across different climate treatments for each species, period and soil type. Greek symbols indicate significant differences across the different drought-rewetting-drought periods for each species, climate treatment and soil type. Asterisks indicate significant differences between acidic and calcareous soil within each climate treatment, species and time course (*P* ≤ 0.050).

**Fig 5:**
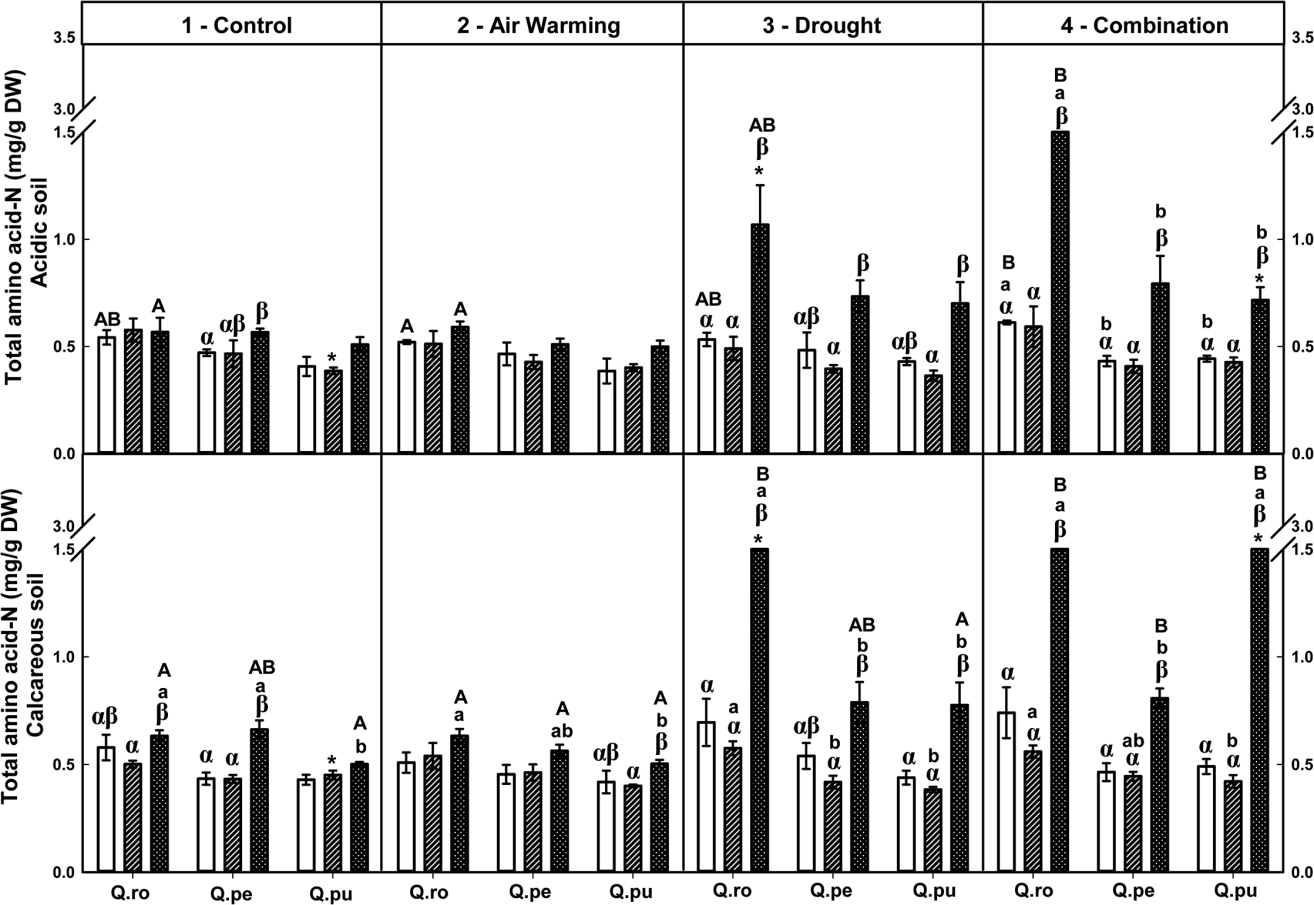
Foliar total amino acid-N of three oak species exposed to different climate treatments and soil types during a drought-rewetting-drought course. Vertical bar charts show mean and standard error and the bar charts were grouped for each species, in each treatment and soil type. Periods are indicated from left to right side: white bar = the first drought period (1^st^D), hedged bar = the rewetting period (RW), cyclone bar = the second drought period (2^nd^D). Different small letters indicate significant differences across three oak species within each climate treatment, period and soil type. Different capital letters indicate significant differences across different climate treatments for each species, period and soil type. Greek symbols indicate significant differences across the different drought-rewetting-drought periods for each species, climate treatment and soil type. Asterisks indicate significant differences between acidic and calcareous soil within each climate treatment, species and time course (*P* ≤ 0.050).

**Fig 6:**
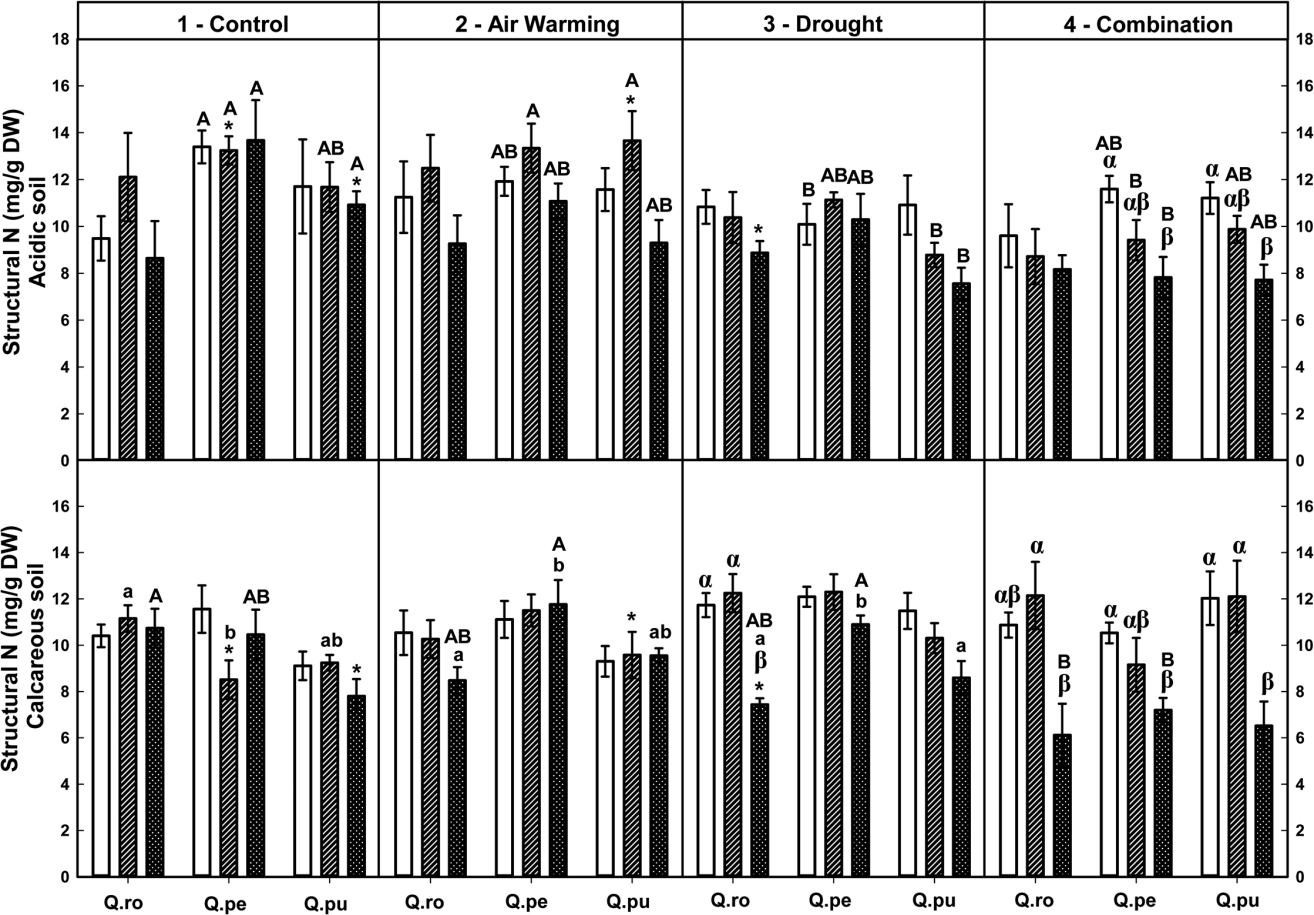
Foliar total structural N of three oak species exposed to different climate treatments and soil types during a drought-rewetting-drought course. Vertical bar charts show mean and standard error and the bar charts were grouped for each species, in each treatment and soil type. Periods are indicated from left to right side: white bar = the first drought period (1^st^D), hedged bar = the rewetting period (RW), cyclone bar = the second drought period (2^nd^D). Different small letters indicate significant differences across three oak species within each climate treatment, period and soil type. Different capital letters indicate significant differences across different climate treatments for each species, period and soil type. Greek symbols indicate significant differences across the different drought-rewetting-drought periods for each species, climate treatment and soil type. Asterisks indicate significant differences between acidic and calcareous soil within each climate treatment, species and time course (*P* ≤ 0.050).

**Fig 7:**
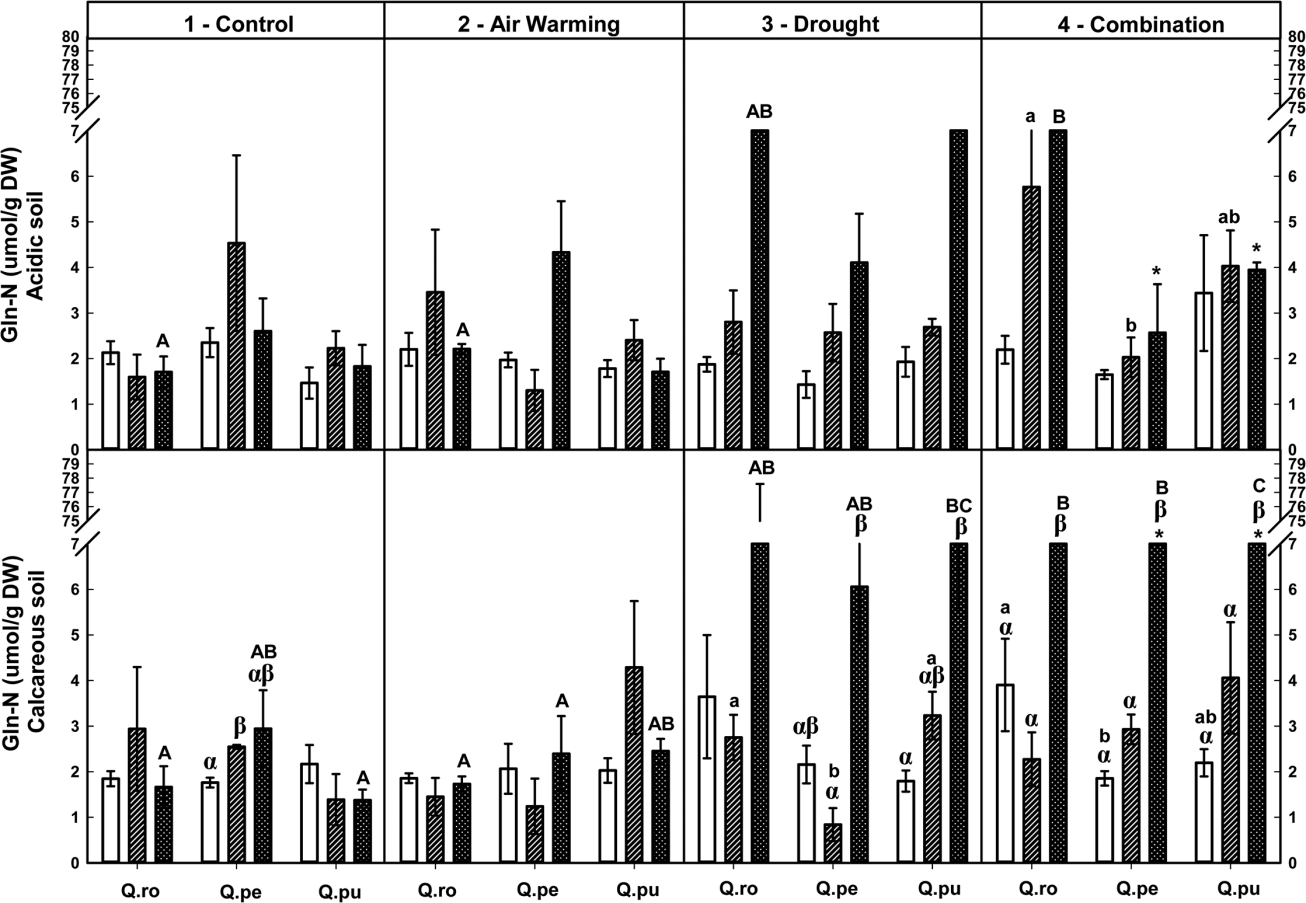
Foliar Glutamine (Gln)-N of three oak species exposed to different climate treatments and soil types during a drought-rewetting-drought course. Vertical bar charts show mean and standard error and the bar charts were grouped for each species, in each treatment and soil type. Periods are indicated from left to right side: white bar = the first drought period (1^st^D), hedged bar = the rewetting period (RW), cyclone bar = the second drought period (2^nd^D). Different small letters indicate significant differences across three oak species within each climate treatment, period and soil type. Different capital letters indicate significant differences across different climate treatments for each species, period and soil type. Greek symbols indicate significant differences across the different drought-rewetting-drought periods for each species, climate treatment and soil type. Asterisks indicate significant differences between acidic and calcareous soil within each climate treatment, species and time course (*P* ≤ 0.050).

**Fig 8:**
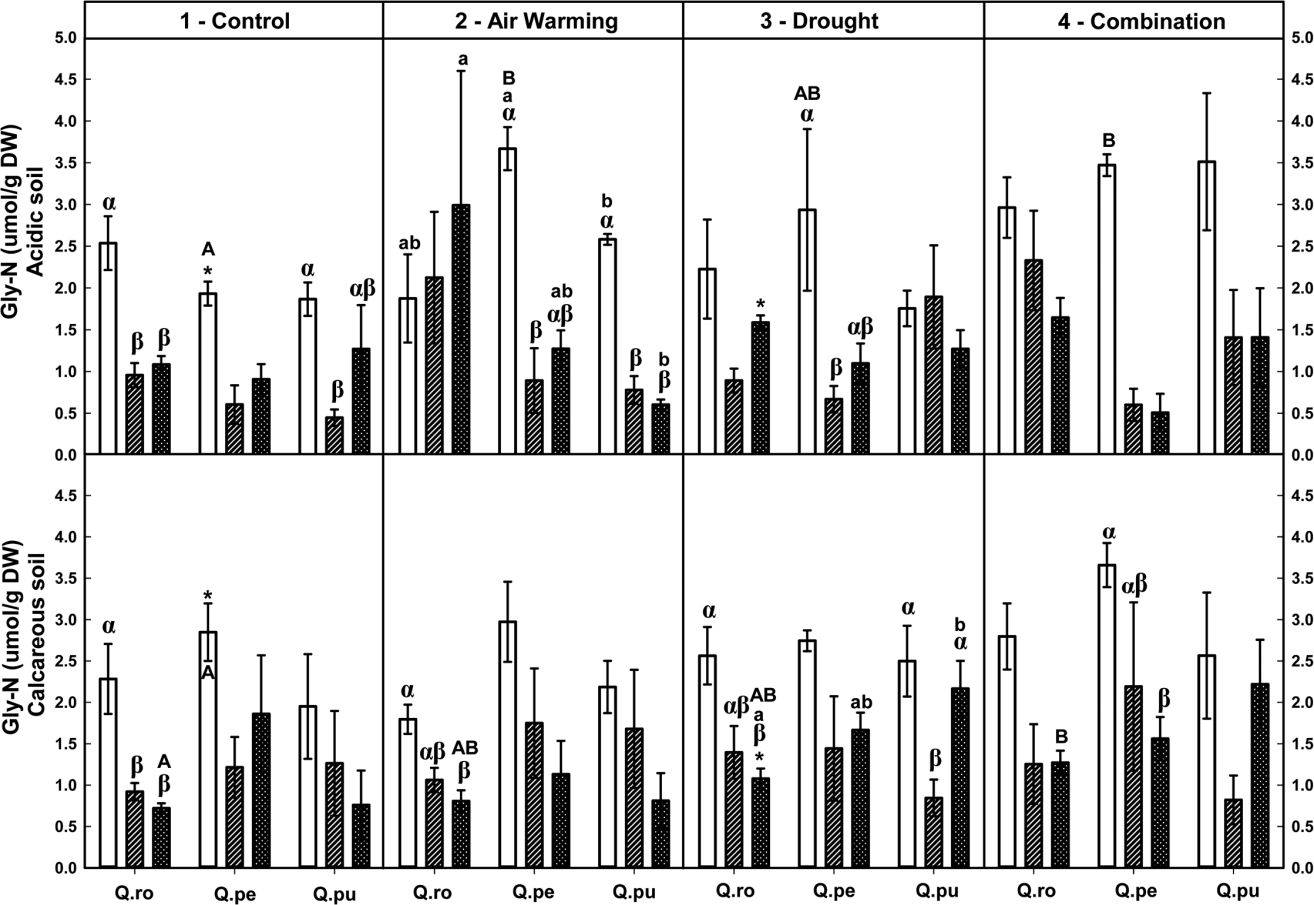
Foliar Glycine (Gly)-N of three oak species exposed to different climate treatments and soil types during a drought-rewetting-drought course. Vertical bar charts show mean and standard error and the bar charts were grouped for each species, in each treatment and soil type. Periods are indicated from left to right side: white bar = the first drought period (1^st^D), hedged bar = the rewetting period (RW), cyclone bar = the second drought period (2^nd^D). Different small letters indicate significant differences across three oak species within each climate treatment, period and soil type. Different capital letters indicate significant differences across different climate treatments for each species, period and soil type. Greek symbols indicate significant differences across the different drought-rewetting-drought periods for each species, climate treatment and soil type. Asterisks indicate significant differences between acidic and calcareous soil within each climate treatment, species and time course (*P* ≤ 0.050).

Foliar soluble protein-N and total amino acid-N concentrations were increased by the D or AWD treatment at the end of the 2^nd^D period on calcareous soil (Table 2, Figs 4 and 5), whereas potassium tended to decrease (Table 2, S4 Fig). Additionally, concentrations of both N fractions were increased by AWD compared to single D or AW treatment at the end of the 2^nd^D period (*P* ≤ 0.048, Figs 4 and 5). At the end of the 2^nd^D period, foliar structural N concentrations were decreased by drought compared to the controls (Table 2, Fig 6). All specific free amino acid-N concentrations in the leaves were increased in the D as well as AW (except AW for glutamine-N and serine-N) treatment over the drought-rewetting-drought course on both soil types (*P* ≤ 0.044, Table 2, Figs 7 and 8, S1–S3 Figs). In particular, glutamine (Gln)-N and serine (Ser)-N concentrations of *Qro* and *Qpu* leaves were increased with D and AWD compared to the control and AW treatment in both soil types at the end of the 2^nd^D period (*P* ≤ 0.044, Fig 7 and S3 Fig), whereas no effects were observed at the end of the 1^st^D period and after RW (Fig 7).

### Variation in foliar N metabolites during the drought-rewetting-drought course

Foliar soluble protein-N and amino acid-N concentrations were generally increased at the end of the 2^nd^D period compared to the 1^st^D period and RW, particularly in the D and AWD treatment. As a tendency their concentrations were lower after rewatering (Table 2, Figs 4 and 5). Calculated structural N concentrations were decreased accordingly (Table 2, Fig 6). Also foliar Gln-N concentrations were significantly higher at the end of the 2^nd^D period than the 1^st^D period and RW (RW showed a tendency to lower values) in the D and AWD treatment on calcareous soil (*P* ≤ 0.025, Fig 7), but not Gly-N concentrations (Fig 8). Similar patterns were observed for Ser-N and Asp-N concentrations in *Qro* and *Qpe* leaves in response to D or AWD treatment compared to RW on calcareous soil (*P* < 0.025, S2 and S3 Figs).

### Responses of foliar N metabolites and potassium as related to species and soil type

Over all climate treatments and both soil types foliar soluble protein-N concentration decreased in the order: *Qro* > *Qpe* > *Qpu* at the end of the 1^st^D period and RW (*P* ≤ 0.034), but at the end of the 2^nd^D period only on acidic soil (*P* ≤ 0.007, Fig 4, Table 2). Moreover, foliar total amino acid-N concentrations were highest in *Qro* compared to *Qpe* and *Qpu* in the D or AWD treatment across the drought-rewetting-drought time course (*P* ≤ 0.033, Fig 5); potassium was highest in *Qro* compared to *Qpe* and *Qpu* over all treatments (Table 2, S4 Fig). Also significant differences in particular amino acids among species (Gln, Glu, Table 2, Fig 7 and S1 Fig) depended on the treatments, soil types and periods.

Comparing the two soil types, levels of foliar soluble protein-N, amino acid-N and potassium in all oak species were significantly enhanced on calcareous soil compared to acidic soil after RW and at the end of the 2^nd^D period (*P* ≤ 0.042, Table 2, Figs 4 and 5). Foliar Gln-N in *Qpe* and *Qpu* showed higher levels on calcareous than on acidic soil at the end of the 2^nd^D period in the AWD treatment (*P* ≤ 0.029, Fig 7). Moreover, pooled over oak species, leaf Glu-N, Asp-N and Ser-N concentrations were enhanced on calcareous compared to acidic soil at the end of the 2^nd^D period (*P* ≤ 0.040, S1– S3 Figs).

## Discussion

### Delayed responses in the dynamics of foliar N metabolites and C:N ratio over the drought-rewetting-drought course

Drought led to significantly enhanced values of foliar δ^13^C abundance across the entire time course applied [26] indicating severe drought stress for all oak saplings investigated. The increased leaf total soluble protein-N and amino acid-N concentrations (particularly Gln-N, Asp-N and Ser-N) were observed at the expense of structural N only at the end of the 2^nd^ D period. Thus, the effect of drought on N partitioning in oak leaves was not immediate, but delayed. It reflects the increased need for the synthesis of N containing osmolytic compounds against the drought stress along the consecutive drought-rewetting-drought course. Apparently, this enhanced need for N was not met by the mobilization of leaf external N sources, such as N stored in bark and wood [37], but only at the expense of internal structural N in the three oak species studied. Iincreased total soluble protein-N and amino acid-N concentrations were also found in the control treatment at the end of 2^nd^ D period, but not for leaf total N, structural N concentrations and the C:N ratio. This indicated that the mobilization of an external N pool by well-watered oak trees was enough to counteract any possible phenological effect. Furthermore, an enhanced need for N containing osmolytic compounds may be due to a higher stress severity of the 2^nd^ D period as indicated by the soil water contents, growth rate of shoots, predawn leaf water potential, stomata conductance and net photosynthesis of the leaves [9, 38]. Apparently, osmolytic compounds accumulated during the 1^st^ D period and maintained during the short-recovery phase [26], were not sufficient to counteract drought stress during the 2^nd^ D period. Similar effects along the drought-rewetting-drought course were found for the foliar C:N ratio that increased due to higher leaf C and lower N concentrations at the end of 2^nd^ D period. From these results it appears that drought mediated changes in N partitioning of oak leaves were strongly related to the severity of drought stress.

### Foliar N metabolite partitioning and total C, N and K concentrations response to climate treatments

Drought increased foliar soluble osmolyte-N concentrations as reflected by amino acid accumulation, confirming hypothesis (ii) whereas leaf structural N concentrations of oak saplings were reduced in the drought treatments (i.e. D and / or AWD) compared to well-irrigated plants (AW and controls). This suggests that drought-stressed leaves invested more N resources into organic N solutes as an acclimation strategy under impaired photosynthesis rates [9]. Moreover, accumulation of amino acids in the leaves is commonly considered as a cellular stress response to drought that plays a major role in the osmotic adjustment thereby promoting leaf turgor maintenance and reducing cell damage [39–42]. In many woody species, specific amino acids and cations such as proline (Pro) and/or γ-aminobutyric acid (GABA) and K^+^ Na^+^ are the principal compounds involved in osmotic adjustment under drought [18, 21, 43–44]. The increases in total amino acid-N as well as specific amino acid-N concentrations (i.e. glutamine (Gln)-N, serine (Ser)-N, aspartic acid (Asp)-N, glycine (Gly)-N) in D treated leaves over the drought-rewetting-drought course observed in the present study are consistent with these reports, but together with many other reports (e.g. [24–26,45–46]) show that the accumulation of foliar osmolyte-N compounds under drought is more general and not restricted to the amino acids mentioned above. Thus, the enhanced concentrations of amino acids-N corroborate a close relationship with active osmotic-protection to maintain a positive foliar cell turgor of the investigated oak saplings upon drought stress [47–50]. However, foliar potassium concentration tended to be decreased in the drought treatment contradicting hypothesis (ii). It was enriched on the calcareous *versus* the acidic soil and appeared to rather depend on the soil type than a foliar reaction. The acidic topsoil had a K_exch_ (mg kg^-1^) of 30.7, the acidic subsoil a K_exch_ (mg kg^-1^) of 19.0 compared to the calcareous soil with 24.8 over both layers [30]. Drought shifted root distribution to lower depths [51], which ensured an increased potassium nutrition. The mechanisms favoring either foliar amino acids for osmoprotection against drought (oaks, present study) over cations (K^+^, birch, [52]) may depend on nutrient availability in the soil and species properties.

The lack of air warming induced accumulation of foliar osmolyte-N concentrations in the tested oak saplings in contrast to hypothesis (ii), may be the result of the small increase in air temperature in the present experiment (increase in daily air temperature: approx. +1 to 2°C). A similar lack of effects of a minor increase of air temperature on the foliar N pools of trees was also found in other studies (reviewed by [5, 23, 53–54]). Still, the combination treatment AWD showed a synergistic amplified effect on leaf soluble N composition compared to the single D or AW treatments, comparable to the combined negative effect of this treatments on plant net primary production (NPP) observed in other studies [55–56]. This result clearly shows that the evaluation of potential consequences of climate change requires the combined analysis of different climate factors to identify synergistic, antagonistic, or additive effects [53, 56–57]. Similar combined effects of D and AW were reflected in the present experiment by decreased predawn leaf water potential and net photosynthesis rates under AWD compared to D or AW [9].

Surprisingly, AW caused an enhanced C:N ratios in the leaves of oaks at the end of 2^nd^ D period. Several laboratory studies showed lower leaf N concentrations in plants growing at high compared to low temperatures [58–59]. For instance, for forb (i.e. *Ambrosia psilostachyia*, *Aster ontarionis*, and *Aster ericoides*) and oak species (i.e. *Q*. *robur*) studied in warmer regions, AW quickly inhibited N mineralization processes in the soil resulting in a progressive decrease in foliar N concentrations compared to ambient temperature controls [60–62]. Decreased leaf N concentration and increased C:N ratios were also found in a global survey of over 1000 plant species on the relationship between leaf C and N levels and local climate indices: leaf N levels decreased when mean annual air temperature increased [63]. This effect has been interpreted as evidence for an offsetting mechanism to reduced rates of biochemical reactions caused by the low efficiency of N-rich enzymes at low temperatures [58–59, 64–65], which was obviously in the present study with a relatively small temperature difference not the case.

### Foliar N metabolite partitioning as related to species and soil type

In former studies, *Qro* showed higher water requirements and was more drought- and thermo-sensitive than *Qpe* and *Qpu* [66–67], the latter with a main distribution in dry and warm Mediterranean areas of Southern Europe [6, 68]. In the present study, *Qro* had highest foliar total protein-N, total amino acid-N (including in particular Gln-N, Gly-N, Glu-N) as well as potassium (K^+^) concentrations regardless of climate treatment and soil type. It also showed a significantly impaired photochemical efficiency of PSII (F_V_/F_M_) as a consequence of reduced pre-dawn leaf water potentials under D treatments compared to *Qpe* and *Qpu* [9]. Apparently, the requirements for the synthesis of N containing osmolytic compounds and other important osmolyte (i.e. K) in response to D and AW treatment increased from *Qpu* via *Qpe* to *Qro*. These results are in line with the most xeromorphic leaf structure (higher pilosity, leaf mass per area (LMA), chlorophyll concentration, C/N ratio, lower leaf size and water content) of *Qpu* compared to *Qro* and *Qpe* under D stress in the present experiment [10].

As hypothesized (iii), foliar N dynamics in the studied oak species during drying-rewetting-drying course depended on soil type, indicated by generally enhanced levels of total soluble protein-N, total amino acid-N and potassium in oak leaves on calcareous compared to acidic soil after the 2^nd^D period regardless of climate treatment. Soil type has been identified as an important factor that strongly modifies the effects of the experimental climate treatments [69–72]. Generally, oaks preferentially develop on acidic soil [12, 73], as also indicated by the foliar biomass of *Qro* and *Qpe* in the present experiment [10]. Considering the lower leaf chlorophyll concentrations and lower stand biomass of oaks growing on calcareous compared to acidic soil as well as the enhanced total N, phosphate and/or manganese contents in the leaves of oaks on acidic soil [10, 30, 74], oak saplings developing on calcareous soil in the present study exhibited a stronger foliar N acclimation capability in response to the D and AW treatments than on acidic soil by investing their internal structural N into the synthesis of N containing osmolytes only during the 2^nd^ D period.

## Conclusions

The present results on foliar N metabolites highlight the generally high drought tolerance of oak species, but also the significance of length and intensity of drought events and rewatering as well as species-specific differences. The first drought event was not intense enough to induce significant changes, but the second drought event of the drought-rewetting-drought course applied significantly induced the formation of more soluble protein and amino acids. Apparently, these compounds were increased for osmolytic adjustment in the leaves of all species at the expense of structural N by leaf-internal processes rather than changes in whole-plant distribution of N. The latter is indicated by the generally unchanged total leaf N content of the leaves. Surprisingly, the increase in soluble amino acids was not only attributed to compounds known to be drought responsive such as Pro and GABA, but to a whole set of major proteinogenic amino acids, including also Gln, Gly, Glu, Asp, and Ser. This results indicates general reprogramming of foliar N metabolism in response to drought and requires further analysis of the molecular changes responsible. All three oak species studied showed these responses, but differed considerably in response of intensity. *Qpu* showed a rather lower reaction to drought in the concentrations of osmotic N compounds, but was shown in another study of this experiment to be drought resistant by morphological and photosynthetic acclimation [9–10]. Reaction intensity of foliar N metabolism to the drought-rewetting-drought course was also highly dependent on soil substrate. The better nutrient availability in the calcareous soil induced stronger changes in soluble N compounds. On one hand, K^+^, known for its function for osmotic adjustment was not significantly changed by the D or AW treatment, irrespective of the different N-supply of the two soil types studied. *Qpu*, the species growing naturally under dryer conditions than *Qpe* and *Qro*, showed constitutively (in the control) higher K and C/N concentrations. Thus, for all biochemical parameters studied, interactions between soil and species were shown during the drought-rewetting-drought course and the D ± AW treatment, which have to be taken into account in future studies on the significance of climate change for the performance of oak trees.

## Supporting Information

S1 Fig Foliar Glutamic acid (Glu)-N of three oak species exposed to different climate treatments and soil types during a drought-rewetting-drought course. Vertical bar charts show mean and standard error and the bar charts were grouped for each species, in each treatment and soil type. Periods are indicated from left to right side: white bar = the first drought period (1^st^D), hedged bar = the rewetting period (RW), cyclone bar = the second drought period (2^nd^D). Asterisks indicate significant differences between acidic and calcareous soil within each climate treatment, species and time course (*P* ≤ 0.050).
(EPS)

S2 Fig Foliar Aspartic acid (Asp)-N of three oak species exposed to different climate treatments and soil types during a drought-rewetting-drought course. Vertical bar charts show mean and standard error and the bar charts were grouped for each species, in each treatment and soil type. Periods are indicated from left to right side: white bar = the first drought period (1^st^D), hedged bar = the rewetting period (RW), cyclone bar = the second drought period (2^nd^D). Different small letters indicate significant differences across three oak species within each climate treatment, period and soil type. Different capital letters indicate significant differences across different climate treatments for each species, period and soil type. Greek symbols indicate significant differences across the different drought-rewetting-drought periods for each species, climate treatment and soil type. Asterisks indicate significant differences between acidic and calcareous soil within each climate treatment, species and time course (*P* ≤ 0.050).
(EPS)

S3 Fig Foliar Serine (Ser)-N of three oak species exposed to different climate treatments and soil types during a drought-rewetting-drought course. Vertical bar charts show mean and standard error and the bar charts were grouped for each species, in each treatment and soil type. Periods are indicated from left to right side: white bar = the first drought period (1^st^D), hedged bar = the rewetting period (RW), cyclone bar = the second drought period (2^nd^D). Different small letters indicate significant differences across three oak species within each climate treatment, period and soil type. Different capital letters indicate significant differences across different climate treatments for each species, period and soil type. Greek symbols indicate significant differences across the different drought-rewetting-drought periods for each species, climate treatment and soil type. Asterisks indicate significant differences between acidic and calcareous soil within each climate treatment, species and time course (*P* ≤ 0.050).
(EPS)

S4 Fig Foliar potassium of three oak species exposed to different climate treatments and soil types at the end of 2^nd^D period. Different small letters indicate significant differences across three oak species within each climate treatment and soil type. Different capital letters indicate significant differences across different climate treatments for each species and soil type. Asterisks indicate significant differences between acidic and calcareous soil within each climate treatment and species (*P* ≤ 0.050).
(EPS)
